# Optimization of Plasma-Sprayed CeScYSZ Thermal Barrier Coating Parameters and Investigation of Their CMAS Corrosion Resistance

**DOI:** 10.3390/ma18225114

**Published:** 2025-11-11

**Authors:** Rongbin Li, Keyu Wang, Ziyan Li

**Affiliations:** School of Materials, Shanghai Dianji University, Shanghai 201306, China; lirb@sdju.edu.cn (R.L.); 13723118211@163.com (Z.L.)

**Keywords:** atmospheric plasma spraying, thermal barrier coatings, orthogonal experiment, porosity, bonding strength, CMAS corrosion

## Abstract

Thermal barrier coatings (TBCs) are critical for protecting hot-section components in gas turbines and aero-engines. Traditional yttria-stabilized zirconia (YSZ) coatings are prone to phase transformation and sintering-induced failure at elevated temperatures. This study fabricated CeScYSZ (4 mol% CeO_2_ and 6 mol% Sc_2_O_3_ co-doped YSZ)/NiCrAlY TBCs using atmospheric plasma spraying (APS). A five-factor, four-level orthogonal experimental design was employed to optimize spraying parameters, investigating the influence of powder feed rate, spray distance, current, hydrogen flow rate and primary gas flow rate on the coating’s microstructure and mechanical properties. The resistance to calcium–magnesium–alumino–silicate (CMAS) corrosion was compared between CeScYSZ and YSZ coatings. The results indicate that the optimal parameters are a spray distance of 100 mm, current of 500 A, argon flow rate of 30 L/min, hydrogen flow rate of 6 L/min, and powder feed rate of 45 g/min. Coatings produced under these conditions exhibited moderate porosity and excellent bonding strength. After exposure to CMAS corrosion at 1300 °C for 2 h, the CeScYSZ coating demonstrated significantly superior corrosion resistance compared to YSZ. This enhancement is attributed to the formation of a CaZrO_3_ physical barrier and the synergistic effect of Ce and Sc in suppressing deleterious phase transformations. This study provides an experimental basis for the preparation and application of high-performance TBCs.

## 1. Introduction

Thermal barrier coatings (TBCs) are extensively employed to protect metallic components in gas turbines and aero-engines [1,2,3]. By significantly reducing the surface temperature of hot-section components, TBCs improve overall thermal efficiency [4,5], enhance resistance to oxidation and corrosion, and extend service life. Common fabrication methods for thermal barrier coatings include atmospheric plasma spraying (APS) and electron beam physical vapor deposition (EB-PVD). APS offers several advantages, including applicability to workpieces of various sizes and geometries, low coating porosity, high deposition efficiency, and low thermal conductivity. A typical TBC system comprises a YSZ (6–8 wt.% Y_2_O_3_-partially-stabilized ZrO_2_) ceramic top coat [6,7], an MCrAlY (M = Ni, Co) bond coat that improves adhesion, and a nickel-based superalloy substrate. However, the conventional YSZ topcoat material is susceptible to phase transformation and pronounced sintering during prolonged exposure at temperatures around 1200 °C, resulting in premature coating failure [8,9]. YSZ exhibits four primary crystal structures depending on temperature: cubic (c), tetragonal (t), metastable tetragonal (t′), and monoclinic (m). At atmospheric pressure, YSZ powder can exist in the cubic (c-), tetragonal (t-), or monoclinic (m-) phase. The c-phase exhibits resistance to transformation into the m-phase at elevated temperatures (>1200 °C), thereby mitigating the associated volume expansion, coating cracking, or spallation [10,11]. Furthermore, the dense structure of the c-phase, coupled with its high fracture toughness and hardness, impedes crack propagation and enhances the coating’s resistance to sintering and erosion. The t′-phase represents a transitional structure formed from the c-phase via a diffusion-controlled shear transformation. This phase belongs to the tetragonal crystal system and exhibits structural characteristics intermediate between those of the t-phase and c-phase. In contrast to the direct transformation path to the m-phase, Lughi et al. [12] reported that the apparent activation energy for the t→m transformation is significantly lower than that required for the t′→t+c transformation. Consequently, the t′-phase remains relatively stable during cooling and does not undergo direct martensitic transformation (t→m). Thus, the t′-phase exhibits greater stability and is less prone to phase transformation compared to the t-phase [13]. The presence of the t′-ZrO_2_ phase in XRD patterns indicates that the YSZ powder has melted in the high-temperature plasma flame, confirming its transition to a molten or semi-molten state during spraying. The t→m phase transformation is accompanied by a 3–5% expansion in unit cell volume, generating stresses that lead to coating spallation and failure. Therefore, developing TBC materials that simultaneously possess long-term high-temperature stability (>1200 °C) and excellent thermal insulation properties represents a critical challenge. Research by Hou et al. [14] demonstrated that doping with rare-earth oxides effectively reduces the thermal conductivity of YSZ TBCs while improving their thermal expansion coefficient, high-temperature phase stability, and sintering resistance. Doping ions are employed to stabilize the ZrO_2_ structure. Stabilizing ions such as M^2+^ (Ca, Mg), M^3+^ (rare earths, Sc, In), and Ce^4+^ (which partially reduces to Ce^3+^ at high temperatures) substitute for Zr^4+^ sites, introducing oxygen vacancies into the ZrO_2_ lattice. The formation of these oxygen vacancies is a crucial factor stabilizing the c-phase. According to Wei et al. [15], coatings prepared with 4 mol% CeO_2_ and 6 mol% Sc_2_O_3_ co-doped YSZ exhibited optimal performance. No m-phase formation was observed after annealing at 1500 °C for 120 h. Furthermore, co-doping effectively synergizes the advantages of individual CeO_2_ and Sc_2_O_3_ doping. This approach enhances the material’s sintering resistance while maintaining an appropriate thermal expansion coefficient, thereby addressing the poor sintering resistance associated with single CeO_2_ doping and the low thermal expansion coefficient resulting from single Sc_2_O_3_ doping. Based on these advantages, APS was employed in this study to fabricate CeScYSZ (CeO_2_ and Sc_2_O_3_ co-doped YSZ)/NiCrAlY thermal barrier coatings for investigation.

The quality of plasma-sprayed CeScYSZ coatings is influenced by numerous factors, among which spray parameters are the most critical. Multiple interdependent parameters exist, and their coupling effects significantly impact the coating’s microstructure and properties. Consequently, identifying the optimal spray parameters is crucial for producing high-quality coatings. Sun et al. [16] employed an orthogonal experimental design to optimize the atmospheric plasma spraying process for 8YSZ thermal barrier coatings and successfully identified the parameter set that yielded superior coating performance. The orthogonal experimental design method provides an efficient and cost-effective approach, enabling the simultaneous evaluation of individual process parameters on coating microstructure and properties, as well as the analysis of coupling effects from multi-factor interactions on coating performance. To optimize the preparation process for atmospheric plasma-sprayed CeScYSZ thermal barrier coatings, an orthogonal experimental design was employed in this study. This design was used to systematically investigate the synergistic effects of key process parameters (e.g., powder feed rate, spray distance, and primary gas flow rate) on the coatings’ microstructure, phase composition, and mechanical properties. This approach facilitated the identification of the optimal process parameter combination.

TBCs are predominantly dictated by the structural stability of the ceramic layer. Therefore, a thorough investigation into the microstructural characteristics of CeScYSZ and its degradation mechanisms during service is of considerable importance. Current research identifies high-temperature oxidation, sintering, and calcium–magnesium–alumino–silicate (CMAS) corrosion as the three primary failure mechanisms responsible for TBC performance degradation. Among these, resistance to CMAS corrosion constitutes a critical challenge within the field of high-temperature materials, with direct implications for the future development of the aerospace, aviation, and energy sectors. For instance, Fang et al. [17] compared the CMAS corrosion resistance of double-layer (featuring a mullite-YSZ protective layer) and single-layer plasma-sprayed YSZ coatings following a 4 h exposure to molten CMAS at 1300 °C. Their results revealed penetration depths exceeding 250 μm in the single-layer YSZ coating, whereas no CMAS infiltration was detected in the YSZ layer of the double-layer coating, demonstrating the superior CMAS corrosion resistance of the double-layer structure under identical conditions. Furthermore, Yuan [18] confirmed that co-doping YSZ with CeO_2_ and Sc_2_O_3_ significantly enhances both its resistance to CMAS corrosion and its mechanical properties. CMAS primarily originates from environmental contaminants such as sand, dust, or runway debris, which are typically composed of CaO-MgO-Al_2_O_3_-SiO_2_ mixtures. At elevated temperatures (typically above 1200 °C), these particles melt to form glassy deposits that adhere to the material surface and penetrate inward through coating defects such as pores and cracks. CMAS corrosion induces coating spallation, thereby directly exposing the underlying alloy to hot combustion gases. This exposure can initiate creep, oxidation, and thermal fatigue, ultimately resulting in blade fracture or burn-through.

An orthogonal experimental design was employed in this study to determine the optimal processing parameters. These parameters were then used to fabricate plasma-sprayed CeScYSZ (4 mol% CeO_2_ and 6 mol% Sc_2_O_3_ co-doped YSZ) coatings. The effects of Ce^4+^ and Sc^3+^ co-doping on the crystal structure, phase composition, phase transformations, and CMAS corrosion resistance of the YSZ ceramics were systematically investigated. The CMAS corrosion behaviors of the YSZ and CeScYSZ coatings were compared under identical conditions to evaluate the enhancement in corrosion resistance afforded by CeScYSZ.

## 2. Materials and Methods

### 2.1. Material Preparation

**Figure 1:**
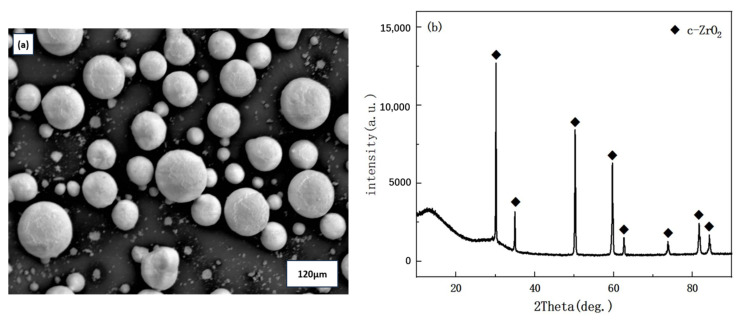
(**a**) Surface morphology of the CeScYSZ powder; (**b**) XRD pattern of the CeScYSZ powder.

NiCrAlY alloy powder was used as the bond coat material. Commercial 8YSZ (Metco 204NS-G, Sulzer Metco Inc., Westbury, NY, USA) and customized 4 mol% CeO_2_ and 6 mol% Sc_2_O_3_ co-doped YSZ ceramic powders were employed as the top coat materials. The morphology and X-ray diffraction (XRD) patterns of the powders are presented in Figure 1. As shown in Figure 1a, the CeScYSZ powder exhibited a spherical and agglomerated morphology with a uniform particle size distribution (30–120 μm), demonstrating satisfactory dispersibility and flowability for plasma spraying. XRD analysis (Figure 1b) revealed that the powder was predominantly composed of the cubic zirconia phase (c-ZrO_2_). Inconel 718 nickel-based superalloy substrates (25 mm in diameter and 5 mm in thickness) were used as the substrate material. Prior to coating deposition, the substrate surfaces were grit-blasted to increase surface roughness and subsequently ultrasonically cleaned in acetone and ethanol for 6 min to remove contaminants.

The CMAS powder was synthesized following the procedure described in Ref. [19]. A powder mixture with a nominal composition of 33 mol% CaO, 13 mol% Al_2_O_3_, 9 mol% MgO, and 45 mol% SiO_2_ was mixed with deionized water and ball-milled for 10 h to achieve homogeneity. The mixture was dried in an oven at 120 °C for 12 h and subsequently heat-treated in a furnace at 1300 °C for 4 h to obtain fully vitrified glass. The resulting CMAS glass was then pulverized using a ball mill. Finally, fine CMAS powder was obtained by sieving through a 500-mesh sieve.

### 2.2. Design of Key Plasma Spraying Parameters

**Figure 2:**
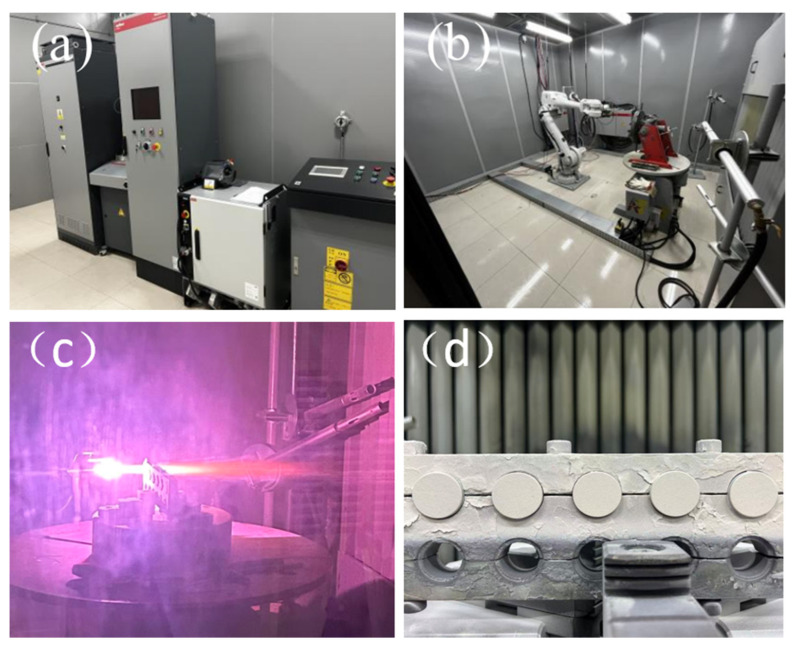
(**a**) Plasma spray control system; (**b**) Spraying chamber; (**c**) Plasma spraying process; (**d**) As-prepared thermal barrier coating sample.

The coating deposition was conducted using an APS system equipped with an F4-MB-6mm torch. The operational parameters were set as follows: The working gas, a mixture of primary and secondary gases, was utilized to generate the plasma. The primary gases, argon (Ar) and nitrogen (N_2_), were ionized to form the main body of the plasma jet. Hydrogen (H_2_) was introduced as the secondary gas to substantially increase the plasma’s enthalpy and thermal conductivity, resulting in a higher-temperature and higher-velocity plume, which consequently enhanced the spraying power and coating quality. The powder carrier gas flow rate was fixed at 2 L/min. The electric current, serving as the core energy input, was applied between the cathode and anode to initiate and sustain the electric arc. The powder feed rate, defined as the mass of spray powder delivered into the plasma stream per unit time, was carefully controlled. Figure 2 illustrates the plasma spraying equipment and the coating preparation process. An orthogonal experimental design was adopted in this study to systematically investigate the coupled effects of five key APS parameters—spray distance, current, primary gas (Ar) flow rate, hydrogen flow rate, and powder feed rate—on the microstructure and mechanical properties of CeScYSZ coatings. Based on a five-factor, four-level orthogonal array (Table 1), other process parameters were held constant using the controlled variable method. The torch traverse speed was set to 1000 mm/s, the step size was fixed at 2 mm, the carrier gas (N_2_) flow rate was maintained at 3 L/min, and the number of coating layers was set to 10. This approach ensured the reliability of the experimental results by eliminating interference from non-studied variables, thus enabling a focused investigation into the synergistic influence mechanisms of the five core parameters on coating performance. The process parameters for spraying the NiCrAlY bond coat are provided in Table 2.

### 2.3. CMAS Corrosion Experiment

**Figure 3:**
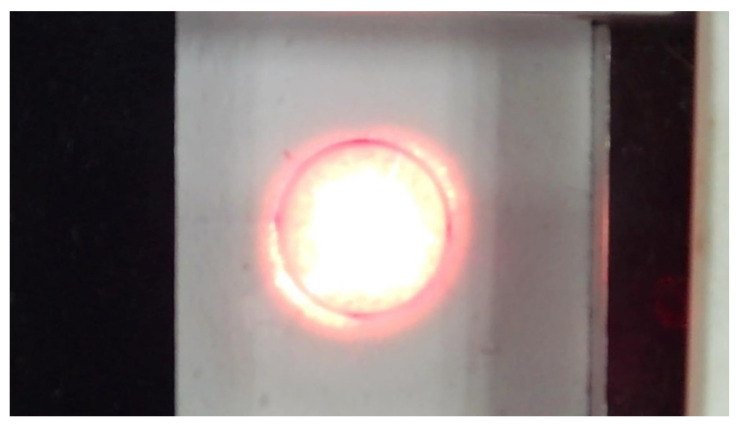
Schematic diagram of the CMAS corrosion test process.

The high-temperature corrosion behavior of TBCs under CMAS molten salt was simulated using a laser thermal shock test (Figure 3). First, CMAS fine powder was mixed with anhydrous ethanol at a ratio of 1 mg per 0.01 mL to form a homogeneous paste. This paste was then uniformly applied to the TBC sample surface at an areal density of 10 mg/cm^2^. After drying at room temperature, CMAS-precoated specimens were obtained. Subsequently, the specimens were placed in a laser thermal shock simulator and heated to 800 °C at a rate of 10 °C/min to initiate CMAS melting. During the experiment, a steady-state temperature gradient was established through precise thermal field control. The coating surface was heated to 1300 °C by a laser to simulate service temperatures, while the alloy substrate temperature was maintained at 800 °C via air cooling. After maintaining this temperature gradient for 2 h, the specimens were cooled to room temperature at a controlled rate of 10 °C/min. This experimental design effectively simulated the penetration behavior of the CMAS melt into the coating under thermo-mechanical coupling fields, thus providing an effective approach for investigating the CMAS corrosion mechanisms of TBCs.

### 2.4. Coating Microstructure and Characterization Techniques

The microstructure and mechanical properties of the coatings were systematically characterized using multiple techniques at different scales. Microstructural observation was performed using scanning electron microscopy (SEM; S-3400N). Phase analysis was carried out via X-ray diffraction (XRD; Rigaku Ultima IV, Bruker, Billerica, MA, USA) using Cu Kα radiation (λ = 1.54178 Å), with a scanning range of 10° to 90° (2θ) and a scanning rate of 10°/min. The cross-sectional porosity was quantitatively characterized through image analysis. Specifically, eight SEM images were acquired at 500× magnification, processed via binarization using Image J software (1.47), and the average pore area percentage was calculated. The bonding strength was measured using a tensile testing machine at a crosshead speed of 1 mm/min. The specimens were prepared according to the Chinese National Standard GB/T 8642-2002 [20]. A CeScYSZ/NiCrAlY bilayer coating was first deposited on Φ25 mm substrate rods. These coated rods were then bonded to counter rods using E-7 high-temperature structural adhesive and dried at 100 °C for 3 h to form test assemblies. This testing protocol enabled a coordinated characterization of the structure-property relationships of the coatings through the integration of multiple techniques. The thermal insulation capability of the coating was characterized using a laser thermal shock testing system. The coating surface was heated by a laser to a stable temperature of approximately 1180 °C, while the temperature variation at the backside of the substrate was recorded. The insulation performance was evaluated based on the temperature difference between the coating surface and the substrate backside.

## 3. Results and Discussion

### 3.1. Influence of Process Parameters on Coating Microstructure

**Figure 4:**
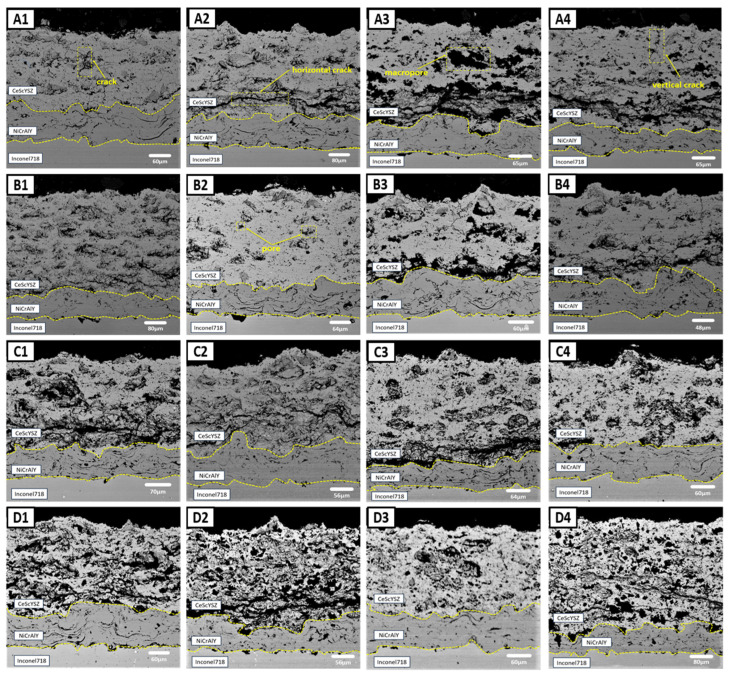
Cross-sectional morphology of 16 coating samples.

The microstructure of the coatings is strongly dependent on the spraying process parameters. Figure 4 shows the cross-sectional morphology of plasma-sprayed CeScYSZ/NiCrAlY thermal barrier coatings produced using different process parameters. The thickness of the CeScYSZ ceramic layers ranged from 240 to 400 μm, indicating that the process parameters significantly influence the coating morphology. The average porosity values of the coatings prepared with the 16 different parameter sets were calculated to be 9.65%, 19.12%, 24.87%, 18.45%, 13.44%, 12.43%, 35.08%, 12.00%, 22.65%, 22.93%, 23.82%, 30.82%, 32.27%, 32.85%, 24.59%, and 30.48%. Within groups A, B, C, and D (grouped by spray distance), the average porosity exhibited minimal variation. However, the average porosity increased significantly with increasing spray distance. This increase is attributed to the attenuation of plasma jet energy, deterioration of particle melting state, and reduced deposition efficiency at greater spray distances, which collectively result in higher coating porosity. Owing to the variations in spraying parameters, the 16 coatings exhibited distinct porosity characteristics. The presence of pores and microcracks can enhance phonon scattering [21,22], reduce thermal conductivity, and consequently improve thermal insulation performance. However, these defects can also provide pathways for the penetration of corrosive media, thereby reducing the service life of the coating. Therefore, optimizing the spraying parameters enables the achievement of an optimal pore structure, thereby enhancing the overall coating performance.

**Figure 5:**
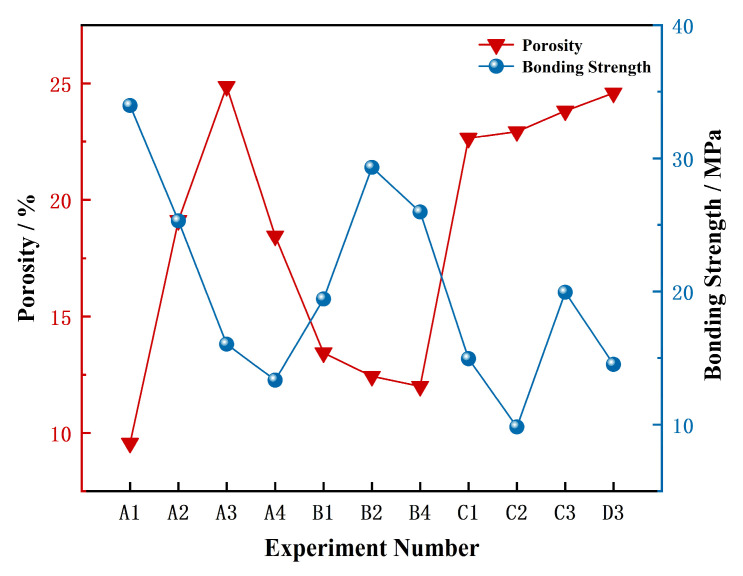
Porosity and bonding strength of CeScYSZ coatings under 11 different spraying parameter sets.

Porosity and bonding strength are critical performance indicators for thermal spray coatings, as they directly govern the coating service life. To quantify the relationship between porosity and bonding strength, we calculated the Pearson product-moment correlation coefficient. The result shows a robust negative correlation with a coefficient (r) of −0.79, as clearly illustrated in Figure 5. This inverse relationship arises because pores occupy volume that would otherwise be available for the formation of solid bonding points, thereby reducing the effective contact area necessary for mechanical anchoring. Since the bonding strength is directly proportional to this effective contact area, a reduction in area inevitably leads to lower strength. Although excessively dense coatings exhibit high bonding strength, they also possess high thermal conductivity (resulting in poor thermal insulation) and increased brittleness. During thermal cycling, such coatings are prone to severe cracking and spallation owing to inadequate stress relief. Conversely, highly porous coatings provide good thermal insulation but exhibit insufficient bonding strength and poor mechanical properties, rendering them susceptible to interfacial delamination during service. Therefore, the design of practical thermal barrier coatings necessitates an optimal balance between adequate bonding strength and appropriate porosity levels to ensure simultaneous thermal insulation and strain tolerance. This study employed range analysis to evaluate the combined effects of the spraying parameters on both the bonding strength and porosity of the coatings. Through normalization, these multiple indicators were transformed into a single comprehensive metric, thereby enabling the optimization of the processing parameters for CeScYSZ thermal barrier coatings.

In range analysis, the sum of the indicator values (e.g., y-values) for all experimental trials corresponding to each level of a given factor is calculated. For example, for level 1 of factor A, the sum of the y-values from all trials conducted at that level is calculated and denoted as K_1_. The average value (k_i_) for each factor level is computed as k_i_ = K_i_/m, where m represents the number of trials conducted at that level. For instance, k_1_ = K_1_/m, where m is the number of replicates per level (i.e., the total number of trials divided by the number of levels for the factor). The range value R for each factor is defined as R = |max(k_i_) − min(k_i_)| and reflects the significance of the factor’s influence on the experimental indicator. A larger absolute R value indicates a greater influence of the corresponding process parameter on the coating performance.

As summarized in Table 3, the order of influence of the process parameters on coating porosity is: spray distance > powder feed rate > argon flow rate > current > hydrogen flow rate. During coating fabrication, the powder feed rate directly determines the coating density and bonding strength by regulating the powder heating process, deposition behavior, and resulting coating structure. Moreover, precise control of the Ar flow rate is equally critical. Insufficient flow leads to uneven droplet spreading and the formation of a porous coating structure, whereas excessive flow reduces the powder residence time in the plasma jet, adversely affecting melting efficiency and potentially promoting pore formation. Therefore, the coordinated optimization of key parameters, particularly the powder feed rate and primary gas flow rate, is essential for obtaining coatings with a dense microstructure and superior performance.

Based on practical considerations and the experimental objective of verifying the coating’s CMAS corrosion resistance, the optimal levels identified via range analysis in Table 2 (those yielding the lowest porosity) were prioritized. To achieve a suitable porosity level, a subset of samples from the orthogonal array was selected using range analysis, guided by these optimal parameters. The selected groups were then subjected to bonding strength testing. The range value (R) was calculated for each factor across its levels. Factors with larger R values indicate that variations in their levels exert a more substantial influence on the experimental outcomes. Particular emphasis was placed on the level combinations of these significant factors, and samples incorporating these key combinations were selected. Simultaneously, experimental groups exhibiting porosity exceeding 30% were excluded. Consequently, 11 groups were ultimately selected from the original set of 16 samples with different process parameters: A1, A2, A3, A4, B1, B2, B4, C1, C2, C3, and D3.

### 3.2. Effect of Process Parameters on Coating Bonding Strength

In APS, high coating bonding strength is crucial for ensuring reliable performance, as it directly determines the mechanical bonding stability with the substrate, spallation resistance, and service life. The bonding performance of coatings is primarily determined by two key factors: the cohesive strength within the ceramic coating itself and the interfacial bonding strength between the coating and the bond coat. Previous studies [23,24,25,26] have demonstrated that significantly enhancing the interfacial bonding strength between the substrate and the coating not only improves adhesion but also effectively prolongs the service life under thermal cycling conditions. This strengthening effect primarily originates from the enhanced interfacial bonding, which better accommodates the thermal expansion mismatch between the coating and the substrate. High bonding strength effectively resists external stresses (e.g., mechanical loads, thermal cycling, corrosive environments), thereby preventing coating cracking or spallation and ensuring the attainment of functional properties such as wear resistance, corrosion resistance, and thermal insulation. Furthermore, bonding strength serves as a critical criterion for optimizing both the spraying process and material selection. Insufficient bonding strength can lead to premature coating failure, potentially resulting in serious failures in demanding applications, such as those in the aerospace and energy sectors. Therefore, enhancing the bonding strength constitutes a central objective in coating design and application.

**Figure 6:**
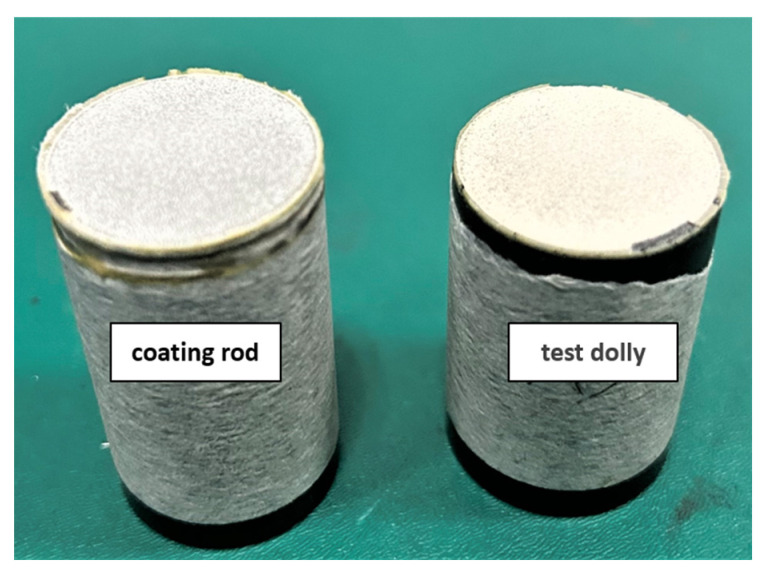
Macroscopic fracture morphology of the CeScYSZ thermal barrier coating after tensile testing.

Figure 6 presents the macroscopic fracture morphology of the CeScYSZ thermal barrier coating following tensile testing. As observed, fracture occurred primarily within the ceramic layer, indicating that the cohesive strength of the coating is lower than its interfacial bonding strength with the bond coat. The key factor influencing the coating’s bonding strength is the internal residual stress. During atmospheric plasma spraying, molten droplets impact the substrate surface and undergo rapid cooling and solidification. The high cooling rate generates significant thermal stresses during droplet solidification and contraction, leading to the formation of microcracks. The nucleation and propagation of these microcracks significantly reduce the overall bonding strength of the coating [27].

As summarized in Table 4, the process parameters influenced the coating bonding strength in the following descending order: hydrogen flow rate > argon flow rate > spray distance > current > powder feed rate. Higher bonding strength increases the coating’s resistance to interfacial delamination and cohesive fracture, consequently significantly enhancing its service life and thermal shock resistance.

In summary, the plasma spraying process involves numerous factors with complex interrelationships, making it difficult to correlate the coating’s microstructure and mechanical properties using single-factor analysis. Therefore, atmospheric plasma-sprayed CeScYSZ thermal barrier coatings must be fabricated under carefully optimized conditions, including spray distance, primary gas flow rate, and powder feed rate. Through orthogonal experimentation, the optimal process parameters were determined as follows: spray distance of 100 mm, current of 500 A, Ar flow rate of 30 L/min, hydrogen flow rate of 6 L/min, and powder feed rate of 45 g/min.

### 3.3. Research on the Thermal Insulation Capacity and CMAS Corrosion Resistance of CeScYSZ Coatings with Optimized Process

**Figure 7:**
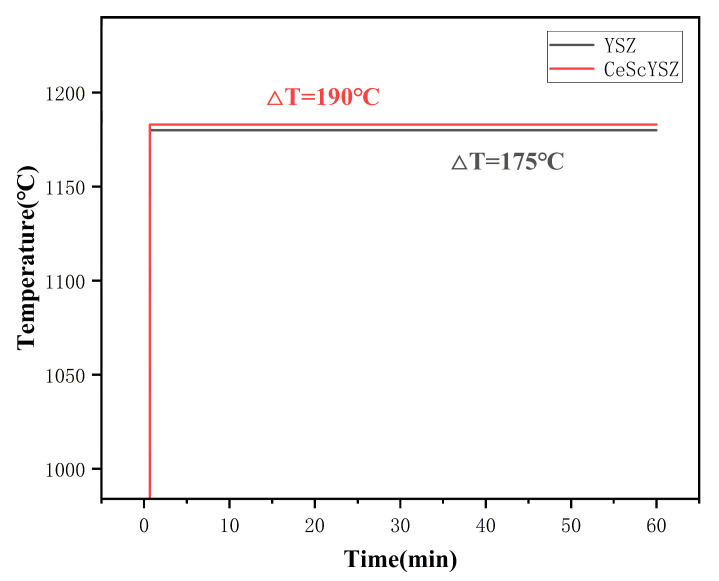
Temperature difference between the surface and substrate versus time for YSZ and CeScYSZ coatings under steady-state conditions.

To evaluate the thermal insulation performance of the modified material, this study compared the steady-state thermal insulation temperature difference between conventional YSZ coatings and CeScYSZ coatings under identical laser thermal shock test conditions (with the coating surface temperature maintained at 1180 °C). The results (shown in Figure 7) demonstrate that the CeScYSZ coating exhibits superior thermal insulation capability.

Specifically, the steady-state thermal insulation temperature difference for the YSZ coating was 175 °C, while that for the CeScYSZ coating reached 190 °C, indicating a notable improvement in thermal insulation performance. This significant enhancement is primarily attributed to the synergistic optimization of the CeScYSZ material system itself and its coating microstructure. Firstly, the co-doping of CeO_2_ and Sc_2_O_3_ introduces a large number of substitutional atoms (Ce^4+^, Sc^3+^, Y^3+^) with varying sizes and masses into the YSZ lattice. This complex lattice distortion significantly enhances the scattering of phonons—the primary carriers of heat conduction—effectively reducing the lattice thermal conductivity, which is the fundamental reason for the improvement in its intrinsic thermal insulation performance. Secondly, the CeScYSZ coating obtained through process optimization was prepared under optimal parameters, resulting in the formation of a more desirable porous structure internally. These pores, microcracks, and unbonded defects between splats act as efficient thermal barriers, further impeding heat flow and enhancing the overall insulation effectiveness of the coating from a macroscopic structural perspective.

In summary, the CeScYSZ material not only reduces intrinsic thermal conductivity at the atomic scale through elemental doping but also constructs an excellent thermal insulation structure at the microscopic scale via appropriate spraying processes, thereby resulting in significantly superior comprehensive thermal insulation performance compared to traditional YSZ coatings.

**Figure 8:**
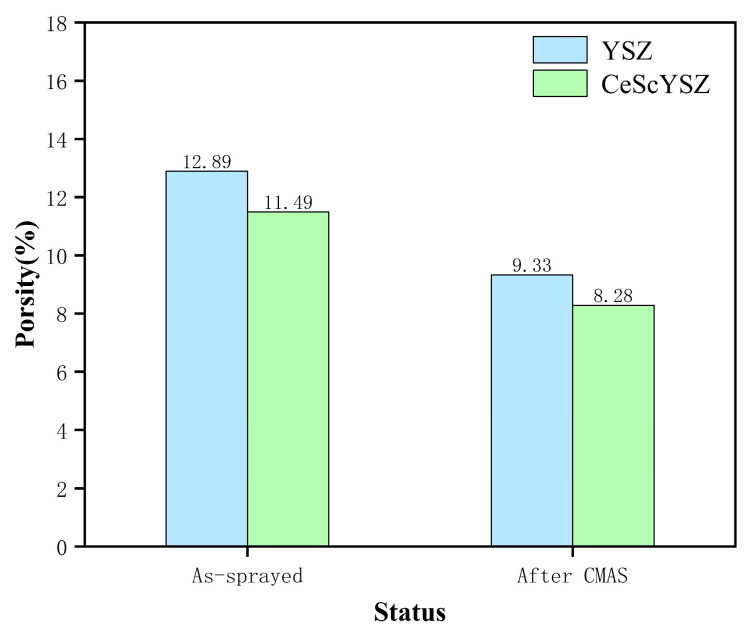
Porosity of YSZ and CeScYSZ Coatings Before and After CMAS Corrosion.

As clearly illustrated in Figure 8, which presents the porosity of both YSZ and CeScYSZ coatings before and after CMAS corrosion, a significant reduction in porosity is observed for both coatings following the 1300 °C heat treatment. This phenomenon is primarily attributed to coating sintering and densification. The high temperature of 1300 °C provides the necessary thermal activation for intense atomic diffusion. This drives mass transport, effectively reducing the high surface energy associated with pores. Consequently, the pores shrink and coalesce, leading to a more compact coating microstructure and the observed notable decrease in overall porosity.

**Figure 9:**
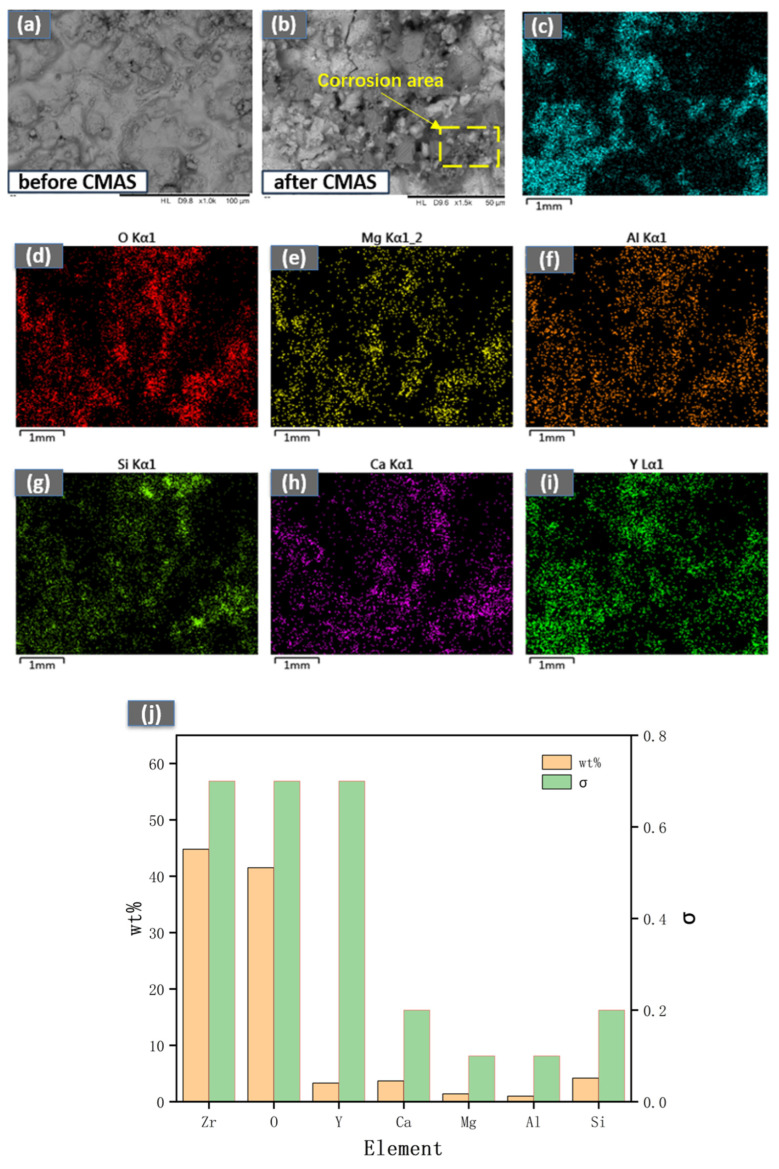
(**a**,**b**) Surface morphology before and after CMAS corrosion; (**c**–**i**) EDS elemental maps of YSZ after 2 h CMAS corrosion at 1300 °C; (**j**) Elemental wt.% on corroded surface.

**Figure 10:**
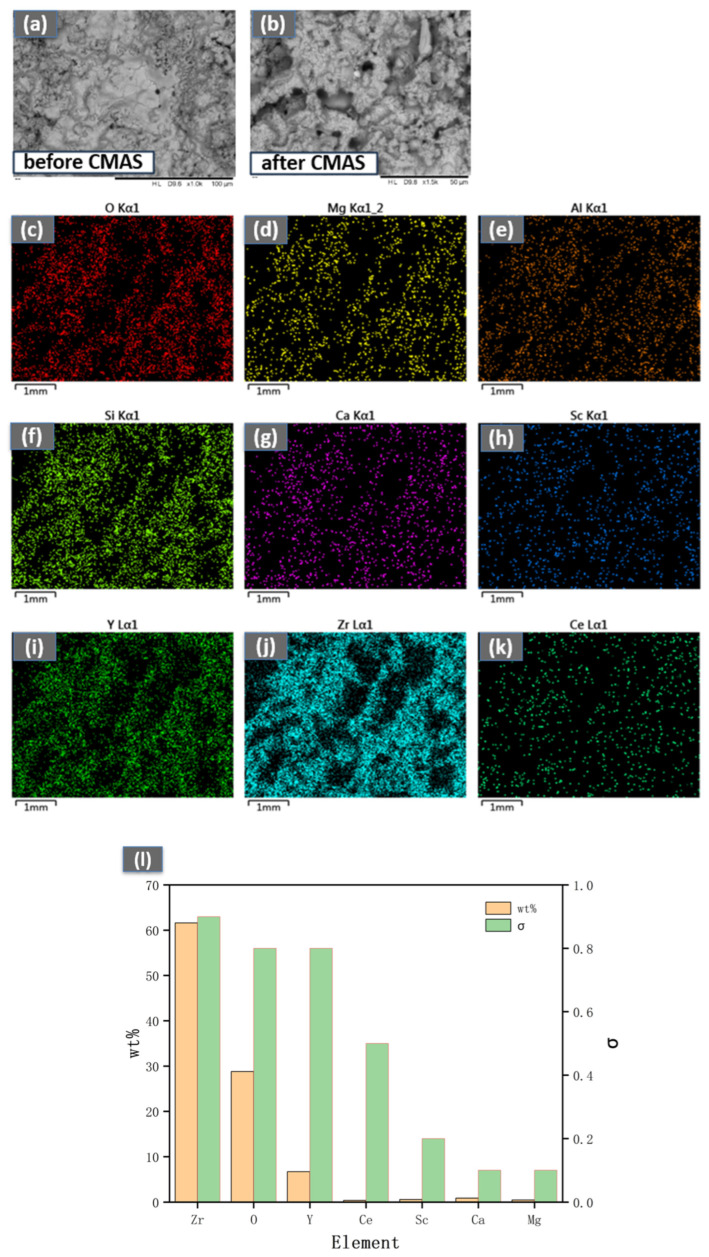
(**a**,**b**) Surface morphology before and after CMAS corrosion; (**c**–**k**) EDS elemental maps of CeScYSZ after 2 h CMAS corrosion at 1300 °C; (**l**) Elemental wt.% on corroded surface.

As shown in Figure 9 and Figure 10, after exposure to CMAS corrosion at 1300 °C for 2 h, significant cracking and the formation of a corrosion product, magnesium silicate (MgSiO_3_), were observed on the surface of the YSZ coating. This compound is thermally unstable at high temperatures and tends to decompose into MgO. The resultant MgO, along with the molten CMAS glass, subsequently infiltrates the coating through cracks and pores. This persistent penetration is difficult to inhibit effectively by the newly formed crystals. In contrast, under identical conditions, the CeScYSZ coating exhibited no similar surface cracking. The CMAS melt primarily infiltrated only the surface layer, where it reacted with the zirconia. The interaction between CeScYSZ and CMAS led to the formation of a calcium zirconate (CaZrO_3_) layer, which acts as a dense physical barrier, effectively hindering further melt penetration. Furthermore, the incorporation of CeO_2_ enhances the oxygen ion conductivity of the coating. This increased conductivity likely promotes the diffusion and transport of species such as Ca^2+^ and O^2−^ at the reaction interface, thereby accelerating the nucleation and growth of the CaZrO_3_ layer. The rapid formation of this barrier consumes calcium from the CMAS, increasing the melt’s viscosity, reducing its fluidity, and ultimately diminishing its penetrating and corrosive capabilities.

**Figure 11:**
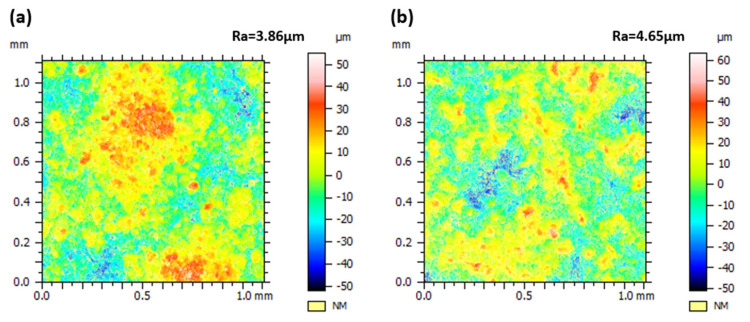
Measuring coating roughness (Ra) with a white light confocal microscope: (**a**) YSZ; (**b**) CeScYSZ.

Figure 11 shows the surface roughness of the coating, as measured by a white light confocal microscope, the APS-fabricated CeScYSZ coating exhibits a higher surface roughness than its YSZ counterpart, attributable to differences in material properties and process parameters. This morphological disparity exerts a markedly different influence on their CMAS corrosion behaviors. For the YSZ coating, the greater surface roughness implies a higher population of surface defects, pores, and microcracks. These features act as direct pathways for CMAS infiltration, significantly increasing the contact area available for detrimental reactions. Consequently, this accelerates the depletion of the stabilizer, dissolution of the ceramic phase, and precipitation of brittle phases, thereby exacerbating corrosion-induced failure. Conversely, for the CeScYSZ coating, its elevated roughness synergizes with its innate high chemical reactivity. The rough surface provides a larger reaction area, promoting a more rapid and extensive reaction with the CMAS melt. This interaction facilitates the generous formation of high-melting-point products, such as CaZrO_3_. These products effectively increase the viscosity of the melt and seal the openings of pores, forming a proactive chemical barrier that halts further CMAS penetration.

**Figure 12:**
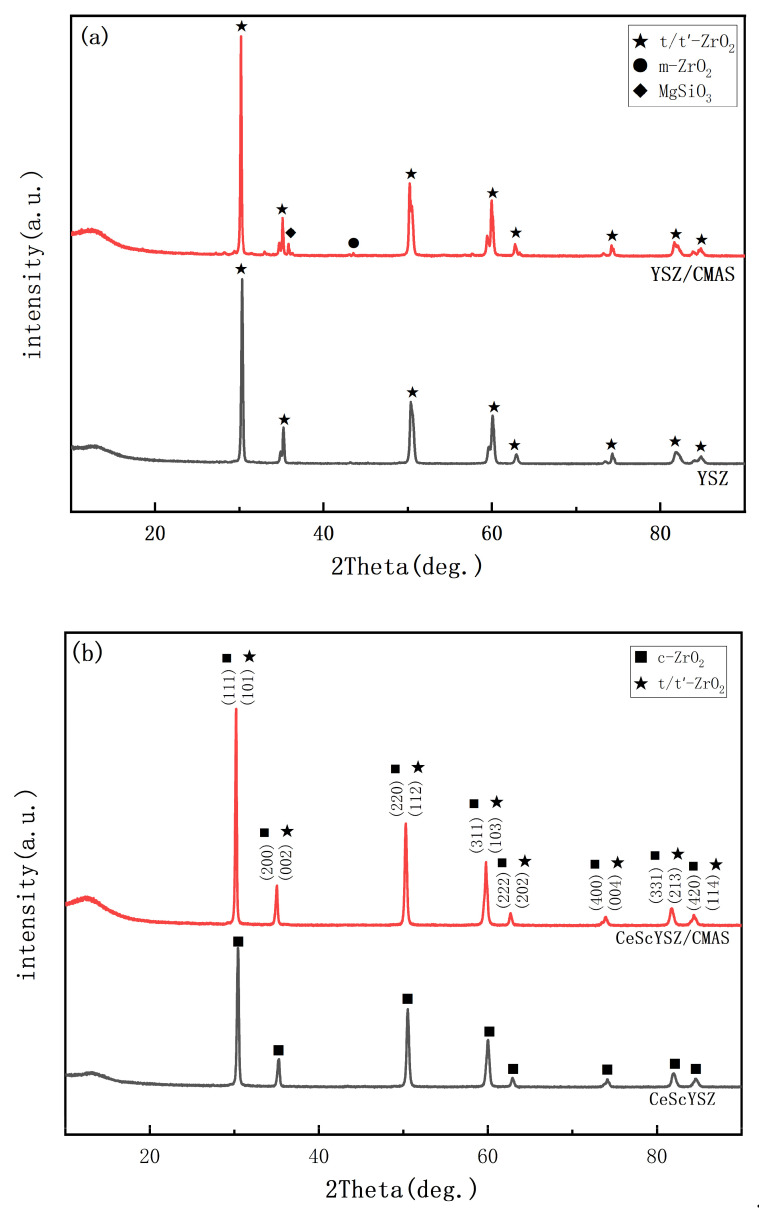
XRD patterns of (**a**) YSZ and (**b**) CeScYSZ before and after CMAS corrosion.

Figure 12a presents the XRD patterns of the YSZ coating before and after CMAS corrosion. The CMAS attack induced a phase transformation in the YSZ, leading to the formation of monoclinic zirconia (m-phase). This transformation is primarily attributed to the dissolution and depletion of yttrium (Y), a vital stabilizer, from the zirconia lattice by the molten CMAS. The consequent Y-depleted regions are unable to retain the metastable tetragonal phase (t’) upon cooling, transforming instead into the more thermodynamically stable monoclinic phase (m). Consequently, the phase stability of YSZ is severely compromised under high-temperature corrosive conditions. Moreover, the associated volume expansion from the t’ to m phase transformation generates substantial internal stresses within the coating. These stresses can initiate microcracks, promote crack propagation, and ultimately result in coating spallation.

Figure 12b presents the XRD patterns of the CeScYSZ coating before and after CMAS corrosion. Notably, no phase transformation was observed following corrosion. The superior resistance is attributed to the higher electronegativity of Ce^4+^, which imparts strong acidity to CeO_2_. This acidity promotes a preferential reaction with the alkaline components (CaO, MgO) in CMAS, forming dense and thermodynamically stable phases such as calcium cerate (CaCeO_3_) or magnesium cerate (MgCeO_3_). This reaction rapidly consumes free Ca^2+^ and Mg^2+^ ions in the melt, effectively blocking its further penetration. Within the CeScYSZ system, Sc and Y serve as stabilizers, transforming ZrO_2_ from a brittle ceramic into a phase-stable material with high toughness. The solubility of Sc^3+^ in CMAS is significantly lower than that of Y^3+^ [28], thereby reducing its propensity to leach out and destabilize the lattice. Furthermore, the ionic radius of Sc^3+^ (0.87 Å) is closer to that of Zr^4+^ (0.84 Å) than that of Y^3+^ (1.02 Å) [29,30]. This closer match enables Sc^3+^ to form stronger bonds with the zirconia lattice, minimizing lattice distortion and enhancing resistance to dissolution. Moreover, the critical doping concentration of Sc_2_O_3_ required to achieve equivalent phase stability (2~3 mol%) is substantially lower than that of Y_2_O_3_ (>4 mol%). Consequently, even with some Sc^3+^ loss, the remaining dopants can effectively sustain the stability of the t-ZrO_2_ phase [31]. These results demonstrate that the CeScYSZ coating exhibits enhanced t-phase stability and superior CMAS corrosion resistance compared to conventional YSZ coatings.

## 4. Conclusions

(1) An orthogonal experimental design with five factors and four levels was implemented to optimize the process parameters for depositing the CeScYSZ coating, using porosity and bonding strength as the key response criteria. The final optimized parameters were determined as follows: spray distance, 100 mm; current, 500 A; primary gas (Ar) flow rate, 30 L/min; hydrogen (H_2_) flow rate, 6 L/min; and powder feed rate, 45 g/min.
(2) The CeScYSZ coating demonstrates enhanced thermal insulation capability relative to the conventional YSZ coating. During CMAS attack, the melt dissolves Y from YSZ, inducing a t→m transformation that creates a porous m-phase layer. Concurrently, MgO infiltrates through cracks, enhancing CMAS penetration. Thermal stress causes significant cracking. In contrast, CeScYSZ forms a barrier layer of CaZrO_3_ that inhibits melt infiltration. Ce^4+^ consumes alkaline ions, aiding protective layer formation, while Sc^3+^ effectively stabilizes the t-ZrO_2_ lattice even in depleted zones. Crucially, the Ce–Sc synergy inhibits Y^3+^ dissolution, entirely blocking the transformation pathway. Thus, CeScYSZ exhibits superior CMAS resistance and phase stability over YSZ.

## Figures and Tables

**Table 1:** Orthogonal experimental matrix of process parameters for plasma-sprayed CeScYSZ coatings.

Exp.	Spray Distance	Current	Ar	H_2_	Powder Feed Rate
	(mm)	(A)	(L/min)	(L/min)	(g/min)
A1	90	500	30	6	35
A2	90	550	35	8	40
A3	90	600	40	10	45
A4	90	650	45	12	50
B1	100	500	35	10	50
B2	100	550	30	12	45
B3	100	600	45	6	40
B4	100	650	40	8	35
C1	110	500	40	12	40
C2	110	550	45	10	35
C3	110	600	30	8	50
C4	110	650	35	6	45
D1	120	500	45	8	45
D2	120	550	40	6	50
D3	120	600	35	12	35
D4	120	650	30	10	40

**Table 2:** NiCrAlY Bond Coat Spraying Parameters.

Stride (mm)	N_2_ (L/min)	Number of Coating Layers	Spray Distance (mm)	Current (A)	Ar (L/min)	H_2_ (L/min)	Powder Feed Rate (g/min)
4	3.5	3	90	600	46	6	29

**Table 3:** Analysis of orthogonal experimental results for porosity.

Exp.	Spray Distance	Current	Ar	H2	Powder Feed Rate	Porosity
	(mm)	(A)	(L/min)	(L/min)	(g/min)	(%)
A1	90	500	30	6	35	9.65
A2	90	550	35	8	40	19.12
A3	90	600	40	10	45	24.87
A4	90	650	45	12	50	18.45
B1	100	500	35	10	50	13.44
B2	100	550	30	12	45	12.43
B3	100	600	45	6	40	35.08
B4	100	650	40	8	35	12
C1	110	500	40	12	40	22.65
C2	110	550	45	10	35	22.93
C3	110	600	30	8	50	23.82
C4	110	650	35	6	45	30.82
D1	120	500	45	8	45	32.27
D2	120	550	40	6	50	32.85
D3	120	600	35	12	35	24.59
D4	120	650	30	10	40	30.48
Range (R)	−12.03	−7.59	−8.08	−5.57	−9.54	
Optimal Level	90	500	30	12	35	

**Table 4:** Analysis of orthogonal experimental results for bonding strength.

**Exp.**	**A1**	**A2**	**A3**	**A4**	**B1**	**B2**	**B4**	**C1**	**C2**	**C3**	**D3**
	**Number**										
**Bonding**	33.96	25.31	16.04	13.34	19.44	29.32	25.97	14.95	9.83	19.94	14.53
	**Strength (MPa)**										
	**Spray Distance** **(mm)**		**Current** **(A)**		**Ar** **(L/min)**		**H_2_** **(L/min)**		**Powder Feed Rate (g/min)**		
**Range (R)**	10.38		5.94		16.15		18.86		5.11		
**Optimal Level**	100		500		30		6		45		

## Data Availability

The original contributions presented in this study are included in the article. Further inquiries can be directed to the corresponding author.
